# Patterns of care in pancreatic cancer radiotherapy: impact of facility volume on outcomes

**DOI:** 10.3389/fonc.2025.1654223

**Published:** 2025-12-05

**Authors:** Arvind Rajan, Elizabeth M. Gleeson, Veronica Pham, Karyn A. Goodman

**Affiliations:** 1 Department of Surgery, University of North Carolina School of Medicine, Chapel Hill, NC, United States; 2 Department of Radiation Oncology, Icahn School of Medicine at Mount Sinai, New York, NY, United States

**Keywords:** radiotherapy, pancreatic cancer, stereotactic body radiotherapy (SBRT), facility volume, overall survival (OS)

## Abstract

**Objectives:**

Radiotherapy (RT), particularly stereotactic body radiotherapy (SBRT), is a promising treatment for borderline resectable or locally advanced pancreatic cancer (PC). This study evaluated the association of RT facility volume with overall survival (OS) and examined patterns of care for patients with PC.

**Methods:**

A retrospective cohort study using National Cancer Database (NCDB) data (2004-2019) included 17,053 PC patients treated with RT, excluding those with metastatic or stage IV disease. RT facility volumes were categorized as low- (<10 cases/year), intermediate- (10–20 cases/year), and high-volume (>20 cases/year). Predictors of OS, including facility volume, RT type, and patient/treatment factors, were evaluated.

**Results:**

Among 17,053 patients (median age 67 years, 51.4% male), 17.6% received SBRT, and 27.1% were treated at high-volume centers. Treatment at high-volume centers (HR 0.863, p<0.001) and receiving SBRT (HR 0.869, p<0.001) were associated with improved OS. SBRT use increased from 8.5% (2004-2011) to 30% (2016-2019).

**Conclusion:**

High-volume RT centers are associated with significantly better survival outcomes in PC patients, particularly those receiving SBRT. These findings emphasize the need to expand access to high-volume centers and develop standardized treatment protocols to improve care and outcomes for all patients.

## Introduction

Pancreatic cancer (PC) has one of the highest mortality rates of any cancer, with an estimated 5-year survival rate of only 11.5% and a complete surgical resection provides the only chance for cure (1). The role of radiotherapy (RT) in the management of PC remains controversial. However, in cases where the tumor is found to be borderline resectable (BRPC) or locally advanced (LAPC), since complete resection with negative surgical margins (R0) may not be possible, emerging evidence suggest a potential role for RT with the aim of improving negative margin rates or reducing the burden of local disease and enhancing local control when surgery is not possible. Neoadjuvant chemoradiotherapy has recently been shown to improve overall survival (OS) when compared to upfront surgery plus adjuvant chemotherapy in patients with resectable and borderline resectable pancreatic cancer (2–4).

Modern advances in RT delivery, particularly stereotactic body RT (SBRT), allow for the precise delivery of a high radiation dose to the target, steep dose gradients beyond the target, as well as reduced overall treatment time (5, 6). However, due in part to the novelty of SBRT in the management of PC, guidelines standardizing optimal patient selection for pancreas SBRT, dosing, simulation technique, and volume delineation are lacking (6). In fact, SBRT was included in the multi-institutional Alliance A021501 phase II trial of neoadjuvant therapy using FOLFIRINOX combined with SBRT versus FOLFIRINOX alone for patients with BRPC and the R0 resection rate was lower in the SBRT arm (7). This study was also associated with low rates of pancreatectomy (35%) and treatment completion (18%) in the SBRT arm, sharply conflicting with the results of many single institution studies of pre-operative SBRT for BRPC and LAPC (8–10). Incorporating pre-operative SBRT followed by surgery in a national study that was performed at small centers with limited experience using this approach instead of limiting the enrollment to high-volume pancreas cancer centers may have impacted on the outcome of this study.

Treatment at higher-volume radiation oncology centers has been associated with improved OS in patients with various cancers (11–15). This association between OS and high-volume centers has also been established in PC resection, with significantly higher mortality rates in patients treated in low-volume centers (16–18). However, the impact of facility volume on OS in patients with PC that receive RT, particularly SBRT, is not well understood. Additionally, despite expanding evidence on the benefits of SBRT in PC in single-institution studies (8, 9, 19), it remains unknown how practice patterns of radiotherapy in BRPC and LAPC are changing in the United States. Ascertaining this information can better characterize the role of RT in PC, as well as identify variations in treatment that can ultimately be addressed to improve care. In the case of pancreatic cancer, where treatment modalities and their outcomes can vary significantly, an examination of these patterns of care can greatly contribute to our understanding and aid in improving treatment strategies.

## Methods

### The National Cancer Database

The National Cancer Database (NCDB) is a joint project of the Commission on Cancer of the American College of Surgeons and the American Cancer Society (20). It draws from over 1,500 commission-accredited cancer programs, representing 70% of all cancer cases in the US. This study utilizes de-identified data from the NCDB.

### Study population

The subset of the NCDB from 2004–2019 that includes all cases coded for pancreatic cancer were included (n=498,987). Only patients with known vital status and follow-up were included (n=458,750). Inclusion was limited to patients who received RT with a dose and number of fractions consistent with either SBRT or Standard Fractionation (n=36,542), however patients who received hypofractionated ablative RT (6000–7500 cGy over 15–25 fractions) were excluded due to low numbers. All patients with metastatic disease or Stage IV disease were excluded from this study (n=31,231). To better ensure that patients were being treated curatively and not palliatively, only patients receiving chemotherapy were included (n=29,436). Additionally, only patients who received surgery after radiation or did not undergo surgery at all were included (n=17,053).

### Study design and measures

This is a retrospective cohort study. The included variables were race, ethnicity, age, year of diagnosis, facility type, insurance status, facility volume, distance to facility, rurality, Charlsson-Deyo comorbidity score, radiation type, and chemotherapy type. Year of diagnosis was categorized into four groups of four years. Facility type was dichotomized by academic/research program status.

Facility volume was measured by utilizing the yearly average RT cases for each institution. These facilities were categorized into low- (<10 cases/yr), intermediate- (10–20 cases/yr), and high-volume (>20 cases/yr) facilities. This volume stratification was determined utilizing the surgical literature as a reference (18). The RT modalities extracted from the NCDB included SBRT and standard fractionation. SBRT was defined as a total dose between 3000–5000 cGy over 6 or less fractions, and standard fractionation was defined as a total dose between 4500–5600 cGy between 25 and 30 fractions.

### Statistical analysis

Statistical analyses were performed in R using the finalfit and survival packages with a significance level at 0.05. Univariate survival analysis was performed with the log-rank test, to assess each variable’s association with overall survival. Patient, clinical, and treatment variables were selected *a priori*; academic/research program status was excluded from the multivariable analysis due to collinearity with facility volume. Cox regression was used to complete multivariable analysis and to estimate hazard ratios (HR) for overall survival with a significance level at 0.05. Overall survival was plotted using the Kaplan-Meier method.

## Results

### Patient characteristics

This retrospective study included 17,053 patients diagnosed with pancreatic cancer between 2004 and 2019 who received radiation therapy (**Table 1**). The cohort was relatively evenly split between sexes (51.4% male, 48.6% female) and predominantly white (82.6%) and non-Hispanic (92.0%). Median age was 67 at diagnosis. Over half of patients had government insurance (59.8%), received care at a non-academic/research center (52.5%), and the majority lived in a metropolitan area (80.7%). Most patients received standard fractionated radiation (82.4%) and multiagent chemotherapy (57.6%).

**Table 1:** Patient characteristics.

Characteristic	Number	Percentage
Sex		
Male	8765	51.4
Female	8288	48.6
Race		
White	14083	82.6
Black	2281	13.4
Unknown	123	0.7
Other	566	3.3
Ethnicity		
Hispanic	676	4.0
Non-Hispanic	15681	92.0
Unknown	696	4.1
Age		
< 70	10354	60.7
>= 70	6699	39.3
Year of diagnosis		
2004-2007	1734	10.2
2008-2011	4797	28.1
2012-2015	5634	33.0
2016-2019	4888	28.7
Academic/Research program		
Yes	7993	47.5
No	8940	52.5
Insurance status		
None	344	2.0
Private	6227	36.5
Unknown	280	1.6
Government	10202	59.8
Facility volume		
High	4617	27.1
Intermediate	3964	23.2
Low	8472	49.7
Distance to facility		
<= 12.7	7630	44.7
> 12.7	7690	45.1
(Missing)	1733	10.2
Rurality		
Metropolitan	13755	80.7
Rural	315	1.8
Urban	2307	13.5
Radiation type		
SBRT	3008	17.6
Standard Fractionation	14045	82.4
Chemotherapy type		
Yes, Unk type	426	2.5
Single Agent	6803	39.9
Multiagent	9824	57.6
Surgery		
Yes	3703	21.7
No	13350	78.3

### Treatment characteristics

From 2004 to 2019, RT utilization in PC treatment increased; between 2004-2011, there were 6,531 patients treated with RT, and between 2012-2019, there were 10,522 patients treated with RT. Additionally, SBRT utilization increased; between 2004 to 2011, only 8.5% of PC cases were treated with SBRT, however, from 2016 to 2019, 30.0% of PC cases were treated with SBRT. Most patients were treated in low-volume RT centers (49.7%), while intermediate- and high-volume centers treated 23.2% and 27.1% of patients, respectively. SBRT utilization increased across all facility volume categories from 2004–2019, with the most rapid adoption at high-volume centers. Among high-volume facilities, SBRT use rose from 7.7% in 2004–2007 to 45.3% in 2016–2019, compared with increases from 5.9% to 20.4% at low-volume centers and from 5.2% to 30.9% at intermediate-volume centers (**Supplementary Table 1**).

### Univariable survival analysis

In univariable analyses, several factors were significantly associated with survival (**Table 2**). Male sex was associated with slightly improved survival (HR 0.962, p=0.0206). Older age (≥70 years) was associated with worse survival (HR 1.233, p<0.001). More recent year of diagnosis was associated with improved survival (p<0.001). Receiving care at a high-volume center (HR 0.665, p<0.001), an academic center (HR 0.741, p<0.001), not having government insurance (HR 0.806 for private insurance, p<0.001), and living farther from a treatment facility (>12.7 miles, HR 0.898, p<0.001) were also predictive of survival. Compared to standard fractionated radiation, SBRT was associated with improved survival (HR 0.717, p<0.001). Patients who received single-agent chemotherapy (HR 1.728, p<0.001) or did not undergo surgery (HR 2.757, p<0.001) had worse survival.

**Table 2:** Univariable and Multivariable Predictors of Overall Survival.

Variable	Univariable			Multivariable		
	HR	95% CI	*P*	HR	95% CI	*P*
Sex						
Female	1			1		
Male	0.962	0.932-0.994	0.021	0.961	0.927-0.996	0.031
Race						
White	1			1		
Black	1.005	0.958-1.054	0.851	0.911	0.863-0.961	0.001
Other	0.830	0.755-0.914	<0.001	0.831	0.748-0.925	0.001
Ethnicity						
Non-Hispanic	1					
Hispanic	0.823	0.755-0.897	<0.001			
Age						
< 70	1			1		
>= 70	1.233	1.193-1.274	<0.001	1.060	1.016-1.105	0.007
Year of Diagnosis						
2004-2007	1			1		
2008-2011	0.800	0.756-0.846	<0.001	0.870	0.819-0.925	<0.001
2012-2015	0.596	0.564-0.630	<0.001	0.771	0.725-0.820	<0.001
2016-2019	0.461	0.435-0.489	<0.001	0.647	0.604-0.692	<0.001
Academic/Research Program						
Yes	1					
No	1.350	1.307-1.395	<0.001			
Insurance Status						
Private	1			1		
None	1.307	1.162-1.470	<0.001	1.068	0.942-1.212	0.302
Government	1.240	1.198-1.283	<0.001	1.114	1.067-1.162	<0.001
Facility Volume						
Low (<10)	1			1		
Intermediate (10-20)	0.822	0.790-0.857	<0.001	0.967	0.923-1.012	0.146
High (20+)	0.665	0.640-0.692	<0.001	0.863	0.823-0.904	<0.001
Distance to Facility						
<= 12.7	1			1		
> 12.7	0.898	0.868-0.929	<0.001	1.051	1.009-1.094	0.017
Rurality						
Metropolitan	1			1		
Rural	1.206	1.072-1.357	0.002	1.099	0.962-1.255	0.166
Urban	1.099	1.048-1.152	<0.001	0.999	0.946-1.055	0.968
Charlsson-Deyo						
0	1			1		
1+	1.046	1.010-1.082	0.011	1.071	1.031-1.112	<0.001
Radiation Type						
Standard Fractionation	1			1		
SBRT	0.717	0.686-0.750	<0.001	0.869	0.827-0.914	<0.001
Chemotherapy Type						
Multiagent	1			1		
Single Agent	1.728	1.672-1.786	<0.001	1.409	1.354-1.466	<0.001
Surgery						
Yes	1			1		
No	2.757	2.639-2.879	<0.001	2.467	2.348-2.593	<0.001

### Multivariable survival analysis

In multivariable survival analysis, many factors remained independently predictive of survival (**Table 2**). Older age (HR 1.060, p=0.007), and having government insurance (HR 1.114, p<0.001) were predictive of worse outcomes. Male sex (HR 0.961, p=0.031) and more recent year of diagnosis (p<0.001) remained associated with improved survival. Receiving care at a high-volume center (HR 0.863, p<0.001) or receiving SBRT (HR 0.869, p<0.001) remained significant predictors of improved survival. Patients receiving single-agent chemotherapy had worse survival than those receiving multiagent therapy (HR 1.409, p <0.001), and those who did not undergo surgery had worse survival than those who did (HR 2.467, p <0.001).

### Kaplan-Meier survival curves

**Figure 1:**
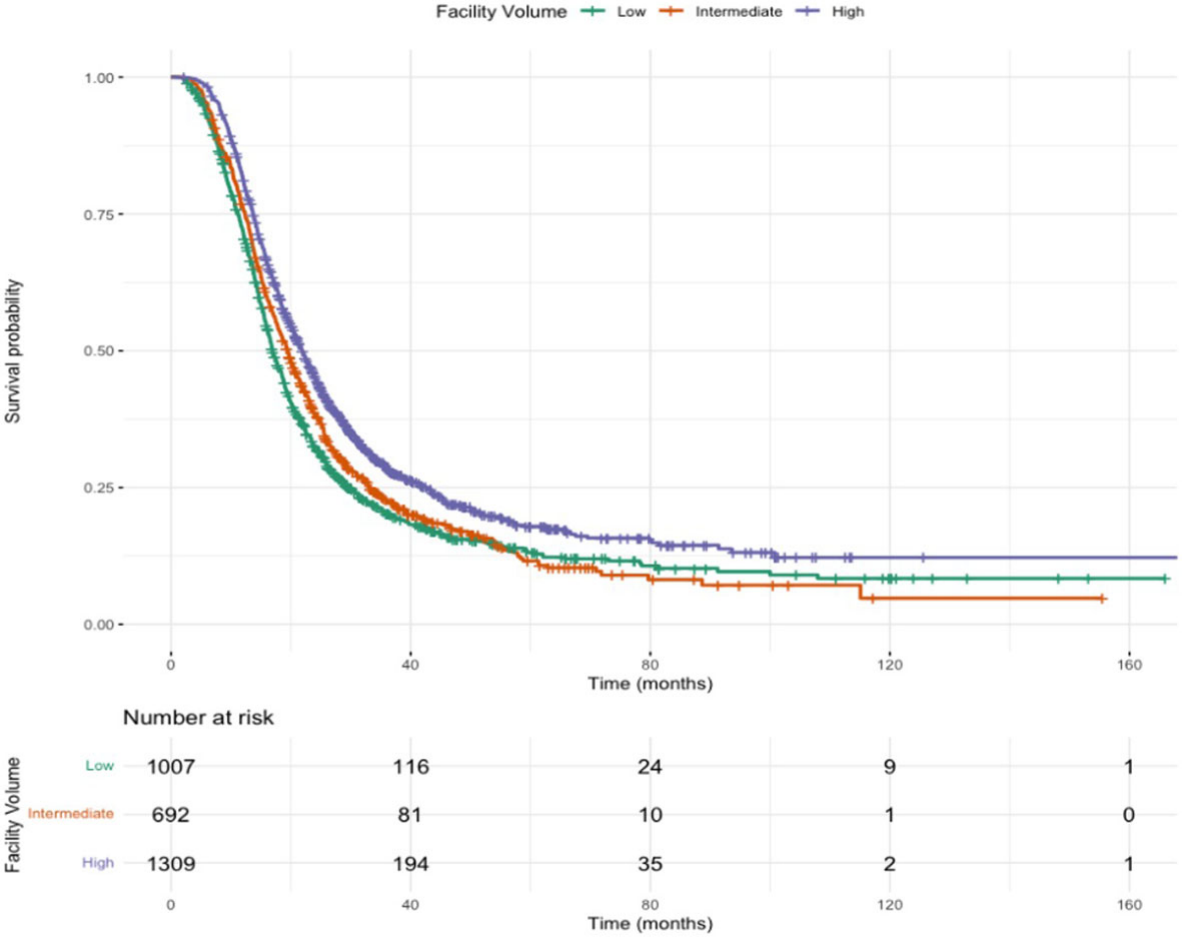
Overall survival for SBRT.

**Figure 2:**
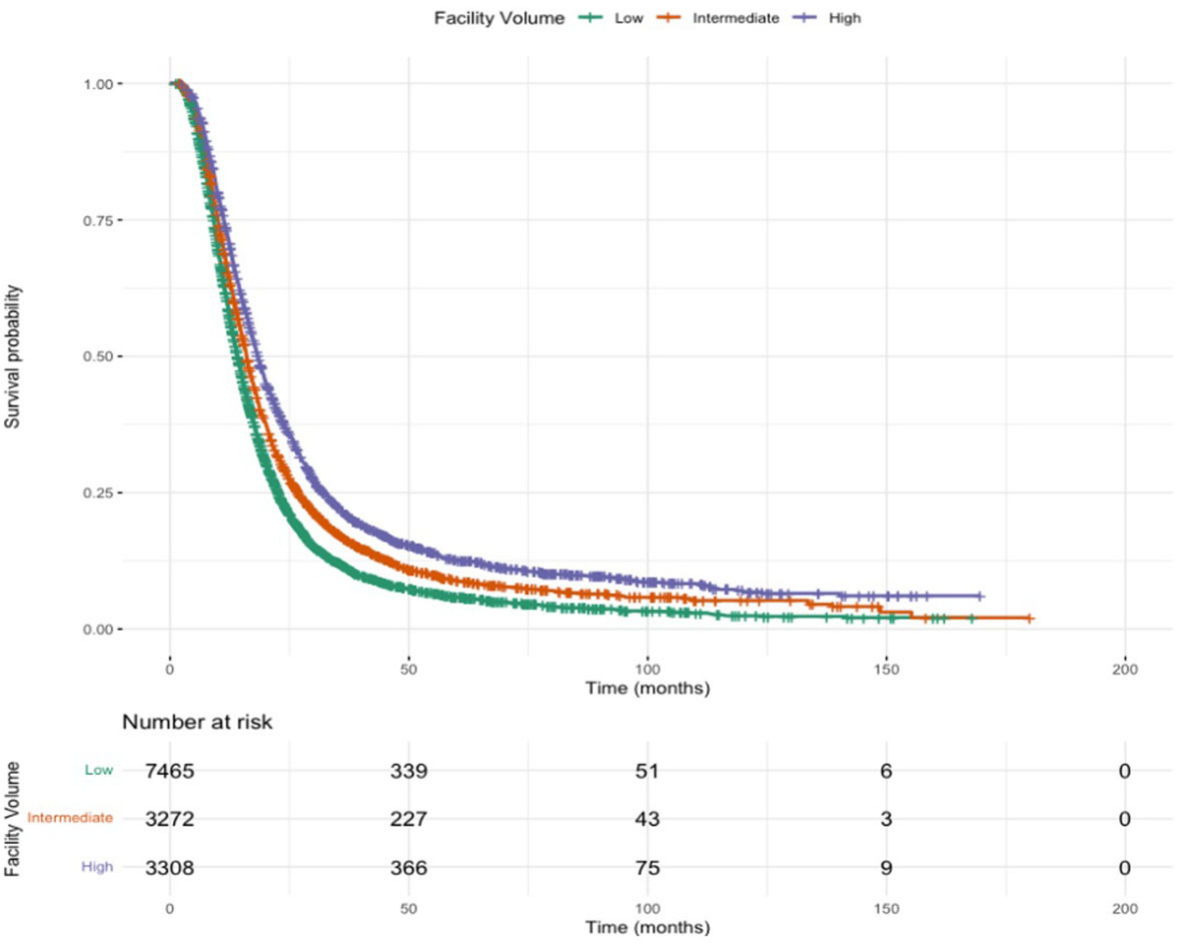
Overall survival for standard fractionation.

Kaplan-Meier survival curves for patients receiving SBRT or standard fractionation in low, intermediate, or high-volume centers are show in **Figures 1** and **2**. Patients receiving SBRT at high-volume centers had significantly higher median survival (21.9 months [20.7-23.2]) than those receiving SBRT at low-volume centers (16.9 months [16.3-18.0]). Additionally, patients receiving SBRT at high-volume centers had significantly higher median survival than those receiving standard fractionation at high-volume centers (21.9 months [20.7-23.2] v. 18.3 months [17.8-19.0]).

## Discussion

While high-volume radiation oncology centers are generally linked to better survival outcomes in various cancers (11–15), the impact of facility volume on PC patients, especially in the context of evolving RT practices for BRPC and LAPC in the U.S., remains poorly understood. This study examined how facility volume affects OS in PC patients receiving RT, particularly SBRT. These findings suggest that high-volume centers are associated with improved OS in patients with LAPC or BRPC treated with RT across the United States, as well as characterize some patterns of care of RT in PC.

High-volume RT centers demonstrated superior OS compared to low-volume RT centers (HR 0.863, p<0.001), consistent with other RT studies examining the association of institutional volume with OS (11, 12). This trend highlights the potential benefits of treatment at high-volume centers, which could be due to factors such as higher rates of compliance with national treatment guidelines, more specialization among radiation oncologists leading to increased experience with tumor identification and contouring, more sophisticated motion management and treatment planning techniques, improved multidisciplinary collaboration, more robust RT quality assurance, and a greater availability of experienced diagnostic, therapeutic, and supportive services (15).

These findings are also consistent with the surgical literature that pancreatic cancer resection at high-volume centers is associated with improved survival outcomes (17, 18, 21). In PC, where resection can be complex due to factors such as anatomical positioning, major blood vessel involvement, and late presentation, underscoring the importance of surgeon experience in navigating these high-risk procedures and the enhanced post-operative care and comprehensive ancillary services in high-volume centers (21). Similarly, the complexity of SBRT for PC, characterized by its novelty, technical and planning sophistication, precise targeting, and lack of guideline standardization emphasizes the role of radiation oncologist expertise. In short, the multidisciplinary team experience and specialization that can be found in high volumes centers likely contribute to improved outcomes in patients receiving complex procedures.

Studies in other cancers such as prostate and lung demonstrate exponential growth in the utilization of SBRT over the past two decades (22, 23). This study suggests a similar increase in the utilization of SBRT in PC; from 2004 to 2011, only 8.5% of PC cases were treated with SBRT, however, from 2016 to 2019, 30% of PC cases were treated with SBRT. When stratified by facility volume, SBRT adoption increased across all center types but was most pronounced at high-volume institutions. SBRT use rose from 7.7% to 45.3% in high-volume centers, compared with 5.9% to 20.4% in low-volume centers and 5.2% to 30.9% in intermediate-volume centers (**Supplementary Table 1**). The slower rise among low- and intermediate-volume centers suggests ongoing disparities in access to SBRT capabilities and experience.

Previous single-institution studies at high-volume centers demonstrate improved OS following neoadjuvant SBRT (8, 9, 19). This study suggests that these findings are consistent among patients treated across the United States, suggesting that there may be improved OS in patients receiving SBRT (HR 0.869, p<0.001) compared to standard fractionation. The Kaplan-Meier curves demonstrate that this trend is not limited to only high-volume centers but is consistent across low- and intermediate-volume centers as well.

Despite the superior OS associated with SBRT, only 17.6% of patients in this study received SBRT, and the majority of these patients were treated in high-volume centers. One explanation could be the extensive multidisciplinary preparation SBRT requires including placing gold fiducial markers endoscopically, high quality treatment planning capabilities, and experienced physicists and radiation therapy staff to implement motion management techniques (24).

The study demonstrated that although low-volume centers utilize SBRT less than high-volume centers, most patients are treated in low-volume RT centers (49.7%). This is a significant finding as it underscores the need to enhance access to treatment options that show significantly improved survival, such as SBRT, in these settings.

In general, our findings suggest that there is a significant increase in OS in patients with PC treated with RT from 2004-2019, across all institutions and treatment types. In addition to more effective chemotherapy regimens such as FOLFIRINOX and gemcitabine/nab-paclitaxel, other contributing factors include advancements in diagnostic techniques, better palliative care, improved chemotherapy and surgery guidelines, as well as advancements in RT such as SBRT.

Despite the large sample size and plethora of variables available through the NCDB, it is limited by its retrospective nature. RT modality was not coded for most patients, hence modality was determined from RT dosing and fractionation. Additionally, the significant underrepresentation of Hispanic patients in our cohort, at only 4.0% versus the national average of 19.1% (25), suggests potential issues in either the NCDB’s ethnicity data collection or access to care at NCDB affiliated centers. This discrepancy is consistent with previous literature, which has documented challenges in accurately capturing ethnicity data from the NCDB, and thus it was excluded from the multivariable analysis (26). Facility-level surgical and radiotherapy volumes are likely correlated in pancreatic cancer, as both treatments are typically concentrated at tertiary centers. While this overlap is unavoidable in real-world data, it should be recognized as a general limitation of national database analyses. The NCDB also does not code for certain factors potentially affecting survival outcomes by radiation facility volume, such as protocol adherence, diagnostic imaging availability, supportive services, and multidisciplinary tumor boards or clinics.

These findings suggest that radiation oncologists’ expertise is critical for providing high-quality RT in PC, especially for SBRT. However, given that most patients receive treatment in low-volume centers, it is crucial to ensure that the high standards of care and treatment protocols established in high-volume centers are consistently implemented in these lower-volume settings.

In conclusion, this study highlights the importance of facility volume in the survival outcomes of PC patients, particularly with the use of SBRT. The increasing adoption of SBRT, its impact on survival, and the current underutilization in lower-volume centers point to the need for broader access to advanced radiation therapies and dissemination of standardized guidelines for contouring, treatment planning and treatment delivery. Educational opportunities should be provided through radiation oncology national societies to ensure that radiation oncologists who may not have training in pancreas SBRT are given the tools they need to incorporate the new techniques into their practices. Incentivizing physicians to pursue further training can be achieved through providing accreditation to radiation oncology sites for specialized techniques such as SBRT based on performance. The observed improvement in survival over time underscores the ongoing advancements in PC treatment, while also indicating areas for future research and guideline development.

## Data Availability

Publicly available datasets were analyzed in this study. This data can be found here: The data that support the findings of this study are available from the National Cancer Database (NCDB).
