# Supplementary material for: Patterns of care in pancreatic cancer radiotherapy: impact of facility volume on outcomes
Source: Front Oncol. 2025 Dec 5;15:1654223. doi: 10.3389/fonc.2025.1654223 (PMC12714612; doi:10.3389/fonc.2025.1654223)
Supplement: Supplementary file 1 [file Table1.docx]

Supplementary Table 1. SBRT utilization over time by facility volume

| **Year of Diagnosis** | **Low Volume (n, %)** | **Intermediate Volume (n, %)** | **High Volume (n, %)** |
| --- | --- | --- | --- |
| 2004–2007 | 60 (5.9%) | 18 (5.2%) | 29 (7.7%) |
| 2008–2011 | 184 (7.4%) | 104 (9.5%) | 163 (13.4%) |
| 2012–2015 | 288 (10.9%) | 209 (15.4%) | 488 (29.8%) |
| 2016–2019 | 475 (20.4%) | 361 (30.9%) | 629 (45.3%) |
